# Dermatoskopie nichtneoplastischer Erkrankungen auf dunkler Haut

**DOI:** 10.1007/s00105-023-05121-w

**Published:** 2023-03-01

**Authors:** Christoph Müller, Harald Kittler

**Affiliations:** Universitätsklinikum für Dermatologie, AKH Wien, Währinger Gürtel 18–20, 1090 Wien, Österreich

**Keywords:** Hauttyp, Inflammatorische Erkrankungen, Pigmentveränderungen, Infektiöse Dermatosen, Dermatoskopische Merkmale, Skin type, Nonneoplastic diseases, Pigmentation disorders, Infectious skin diseases, Dermoscopic characteristics

## Abstract

**Hintergrund:**

Die Dermatoskopie stellt ein wichtiges Hilfsmittel in der allgemeinen Dermatologie dar.

**Ziel der Arbeit:**

Es erfolgt die Darstellung von Unterschieden von heller und dunkler Haut in nichtneoplastischen Erkrankungen mit Fokus auf die Dermatoskopie.

**Material und Methoden:**

Anhand von bisher publizierten Studien sollen dermatoskopische Unterschiede der unterschiedlichen Hauttypen sowie Merkmale von inflammatorischen Erkrankungen und Pigmentveränderungen erläutert werden.

**Ergebnisse:**

Bestimmte Strukturen sind in der Dermatoskopie dunkler Haut schwieriger zu beurteilen (z. B. Gefäße), während sich andere Strukturen (z. B. Follikelöffnungen) prominenter darstellen.

**Diskussion:**

Der Großteil der Studien zur Dermatoskopie stammt aus Studien, in denen vorwiegend Personen mit einem hellen Hauttyp inkludiert wurden. Weitere Studien mit Personen mit einem Hauttyp IV oder höher sind notwendig, um die Diagnostik in dieser Bevölkerungsgruppe zu verbessern.

## Abstract (en)

**Background:**

Dermoscopy is an important tool in general dermatology.

**Objectives:**

To show differences of light and dark skin in nonneoplastic diseases with focus on dermoscopy.

**Materials and methods:**

Using previously published studies, dermoscopic differences of various skin types as well as features of inflammatory diseases and pigmentary changes are illustrated.

**Results:**

Certain structures are more difficult to assess in dermoscopy of dark skin (e.g., vessels), while other structures (e.g., follicular openings) are more prominent.

**Conclusions:**

The majority of studies on dermoscopy are from studies that predominantly included individuals with fair skin types. Further studies of individuals with skin type IV or higher are needed to improve diagnosis in these patients.

Die Dermatoskopie hat sich als wichtiges und effizientes Hilfsmittel in der Dermatoonkologie etabliert. In den letzten Jahren zeigte sich jedoch, dass diese Untersuchungstechnik auch für die Diagnose nichtneoplastischer Hauterkrankungen inklusive entzündlicher und infektiöser Dermatosen sowie bei Pigmentveränderungen wertvoll sein kann. Das dermatoskopische Erscheinungsbild dieser Erkrankungen variiert abhängig vom Hauttyp. Im Vergleich zu heller Haut treten auf dunkler Haut andere dermatoskopische Merkmale in den Vordergrund, und die Kenntnis dieser Unterschiede ist oftmals entscheidend für die richtige Diagnosestellung. Leider besteht nach wie vor ein Überhang an Publikationen zur Dermatoskopie von Hauterscheinungen auf heller Haut sowohl in Hinsicht auf neoplastische als auch auf nichtneoplastische Erkrankungen, während die Beschreibung der Dermatoskopie auf dunkler Haut vernachlässigt wurde. Erst seit geraumer Zeit wurde die Wichtigkeit dieses Themas erkannt und in Publikationen aufgegriffen, die in dieser Übersichtsarbeit zusammengefasst werden.

## Wie unterscheiden sich die unterschiedlichen Hautfarben?

Die aktuell am häufigsten verwendete Einteilung der Hautfarbe ist jene nach Fitzpatrick. In dieser werden 6 Hauttypen unterschieden, wobei Hauttyp I jenen mit dem geringsten und Typ VI jenen mit dem höchsten körpereigenen Sonnenschutz darstellt.

Bei allen Hauttypen ist die Zahl der Melanozyten gleich groß. Unterschiede findet man jedoch in Bezug auf den Melaningehalt, der bei Menschen mit dunkler Haut höher ist. Aber die Menge an Melanin ist nicht der einzige Unterschied. Melanozyten können entweder das braunschwarze Eumelanin oder das gelblich-rote Phäomelanin produzieren. Eumelanin bietet eine deutlich höhere Schutzwirkung vor UV-Strahlung als Phäomelanin, das auch aufgrund der vermehrten Produktion von freien Sauerstoffradikalen mit einem erhöhten Risiko für Hautkrebs einhergeht [14]. Welcher Melaninsubtyp anteilsmäßig vermehrt von den Melanozyten produziert wird, ist genetisch determiniert. So produzieren z. B. Personen, die eine bestimmte Variante im *MC1R*(„Melanocortin-1-Rezeptor“)-Gen aufweisen, einen höheren Anteil an Phäomelanin, was zu einer rötlichen Haarfarbe, einem hellen Teint und hoher UV-Empfindlichkeit führt. Des Weiteren findet man in dunkler Haut tendenziell eine dickere Dermis und kleinere Kollagenfasern [1]. Bei Personen mit afrikanischer Abstammung kommen die Adnexstrukturen deutlich prominenter zur Darstellung. Unterschiede zeigen sich auch hinsichtlich der Hautalterung, die bei dunkler Haut aufgrund des langsameren Abbaus der Melanosomen langsamer verläuft als bei heller Haut [6].

Gewisse Erkrankungen treten deutlich häufiger oder fast ausschließlich bei Personen mit Hauttyp IV bis VI auf. Als Beispiele sind hier die disseminiert rezidivierende Infundibulum-Follikulitis, das Melasma, das Erythema dyschromicum perstans oder der Lichen planus pigmentosus zu nennen.

## Dermatoskopie bei Personen mit dunkler Haut

Die Internationale Gesellschaft für Dermatoskopie (IDS) hat bezüglich der Dermatoskopie nichtneoplastischer Erkrankungen der Haut im Jahr 2020 einen Expertenkonsens veröffentlicht mit dem Ziel, die dermatoskopische Begriffswelt in diesem Bereich zu vereinfachen und zu standardisieren [9]. Ein Jahr später wurde eine Validierung dieses Konsenses auch für die Anwendung auf dunkler Haut publiziert. Man einigte sich auf eine standardisierte Beschreibung, die 5 Attribute umfasst: Gefäße, Schuppung, follikuläre Veränderungen, „andere Strukturen“ und spezifische Merkmale. Innerhalb dieser Kategorien gibt es 31 untergeordnete Elemente, die speziell für dunkle Haut um 5 weitere Subelemente erweitert wurden [7].

Grundsätzlich zeigen sich die meisten dermatoskopischen Kriterien ähnlich, unabhängig vom Hauttyp. Der größte Unterschied findet sich bezüglich der Beurteilung von Gefäßstrukturen. Diese sind bei Personen mit dunkler Haut weniger prominent und somit schwieriger zu beurteilen. Dafür hat bei dunkler Haut die Beurteilung von Adnexstrukturen einen höheren Stellenwert. Die Schuppung kann neben einer weißen oder gelben Farbe zusätzlich auch eine braune Farbe aufweisen, und Bindegewebsreaktionen können in dunkler Haut prominenter zur Darstellung kommen.

Bei dunkler Haut hat die Beurteilung von Adnexstrukturen einen höheren Stellenwert

Dermatoskopisch sieht man in gesunder dunkler Haut dünne braune retikuläre Linien. Histologisch entsprechen diese der pigmentierten Basalschicht. In den Hautfalten können diese auch als parallele braune Linien zur Darstellung kommen. Dazwischen findet man kleine weiße Schollen, die den Ausführungsgängen der Schweißdrüsen entsprechen. Die Gesichtshaut zeigt eine flächige Pigmentierung, die nur durch die runden, hypopigmentierten Öffnungen der Adnexen unterbrochen wird [18].

In weiterer Folge werden Unterschiede in der Dermatoskopie dunkler und heller Haut anhand von ausgewählten Beispielen für inflammatorische und Pigmenterkrankungen dargestellt.

## Inflammatorische Erkrankungen

Die Dermatoskopie hat sich in der Diagnose von inflammatorischen Erkrankungen als ein wichtiges Hilfsmittel in speziellen Situationen erwiesen („Inflammoskopie“). Im Gegensatz zu melanozytären Hautveränderungen stehen hier v. a. die Gefäße, Schuppen, Follikel und Hintergrundfarbe im Vordergrund. Zur Beurteilung der Gefäße ist es empfehlenswert, eine kontaktlose Untersuchung mit polarisiertem Licht durchzuführen, da die Gefäße sonst durch den Anpressdruck kaschiert werden können. Unterschieden werden hierbei: punktförmige, lineare (unregelmäßig oder schlangenförmig), kommaförmige (gebogen), haarnadelartige (geschlungen), glomeruläre (gewunden) und verästelte (schlangenförmig, verzweigt) Gefäße. Des Weiteren kann auch die Anordnung der Gefäße beurteilt werden. Diese können regelmäßig verteilt sein oder aber in Clustern (gruppiert), serpiginös oder randständig angeordnet sein. Der erschwerende Faktor der Dermatoskopie von inflammatorischen Krankheiten bei dunkler Haut ist der höhere Melaningehalt in der Basalschicht, wodurch die Sichtbarkeit der Gefäße eingeschränkt ist. Das führt dazu, dass die charakteristische Rötung entzündlicher Erkrankung auf dunkler Haut fehlen kann. Bezüglich der Schuppung beurteilt man die Farbe und auch deren Anordnung innerhalb einer Läsion.

### Psoriasis

Auch auf dunkler Haut lässt sich die Diagnose Psoriasis schon einfach klinisch stellen. Jedoch gibt es immer wieder diagnostisch schwierige Situationen, v. a. wenn nur wenige Läsionen bestehen oder bei einem atypischen klinischen Bild. Oft kann hierbei die Dermatoskopie hilfreich sein, um den klinischen Verdacht zu erhärten. Hierbei findet man einen geröteten Hintergrund mit regulär angeordneten Punktgefäßen und diffusen weißen Schuppen. Dadurch kann eine Sensitivität und Spezifität jeweils bis zu 88 % erreicht werden. Obwohl die Inzidenz der Psoriasis bei Personen mit heller Haut höher ist, zeigte eine rezente Arbeit aus den USA für Personen mit dunkler Hautfarbe eine Prävalenz von 1,5 % [2]. Bei dunkler Haut findet man im Prinzip die gleichen dermatoskopischen Merkmale wie bei Kaukasiern, jedoch sind sie weniger gut sichtbar, insbesondere die Gefäßveränderungen. In einer Arbeit von Nwako-Mohamadi et al. fand man zum einen, dass die regulär angeordneten Gefäße in dieser Patientengruppe deutlich seltener zu sehen sind, und zum anderen, dass die weiße Schuppung nicht diffus, sondern eher ungleichmäßig verteilt ist. Die klassischen Psoriasis-assoziierten dermatoskopischen Eigenschaften zeigen sich deutlicher im Plaque- als im Patch-Stadium der Erkrankung [15].

### Lichen (ruber) planus

**Figure 1:**
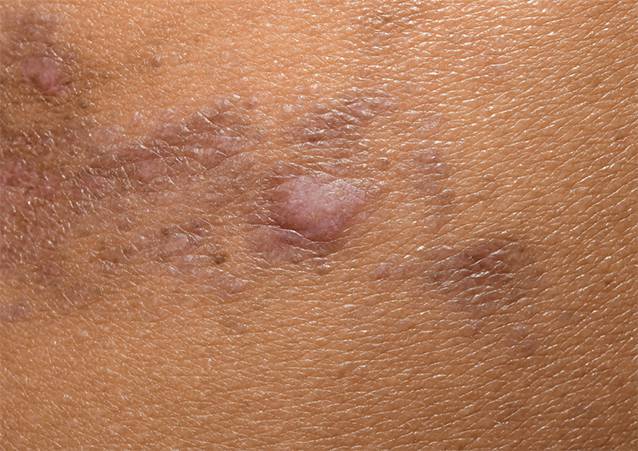

Auch in der Diagnostik des Lichen planus konnte sich die Dermatoskopie als hilfreiches Werkzeug etablieren, wodurch meistens auch auf eine Biopsie verzichtet werden kann. Farblich zeigen sich die Läsionen erythematös bis violett. Mit dem Dermatoskop kommen weiße, kreuzende Linien zur Darstellung, die bei Läsionen in der Schleimhaut klinisch als Wickham-Streifen sichtbar sind (Abb. 1 und 2). Diese Veränderungen sind ein spezifisches Kennzeichen für den Lichen planus [17]. Eher unspezifisch sind die Gefäße, je nach Klinik findet man bei hypertrophen Varianten eher Liniengefäße, bei flachen Läsionen eher Punktgefäße. Bei Personen mit dunkler Haut zeigt sich dieses Zeichen in mehr als 60 % der Fälle. Dies ist vergleichbar mit Personen mit helleren Hauttypen. Im Unterschied zur hellen Haut findet man bei einigen Patienten eine retikuläre Pigmentierung auf einem braunen Hintergrund mit blaugrauen Wickham-Streifen und braunen Schollen [5]. In einer Untersuchung von 55 Läsionen von Patienten mit Hauttyp IV oder dunkler zeigte aber die Mehrheit den klassischen violetten Hintergrund [15]. Die Gefäße sind klassischerweise in der Peripherie angeordnet, können aber in bis zu 80 % der Fälle gar nicht sichtbar sein.

**Figure 2:**
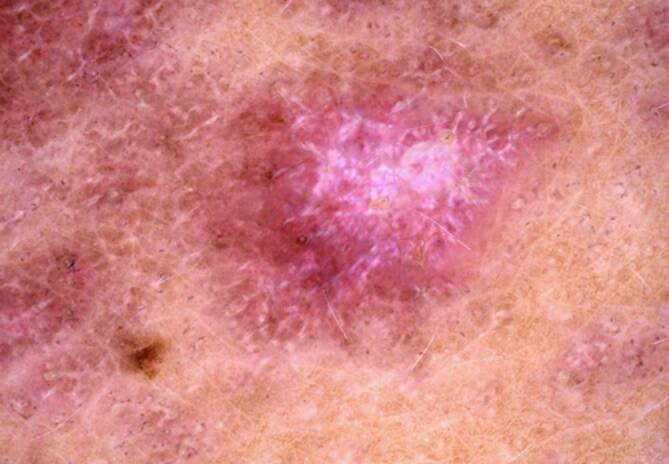

### Pityriasis rosea

Bei der Pityriasis rosea zeigt sich eine charakteristische randbetonte (sog. Collerette-artige) weißliche Schuppung auf einem gelben und rötlichen Hintergrund. Bei Personen mit dunkler Hautfarbe zeigt sich hingegen eine braun-gelbliche Farbe, und wie bei Psoriasis ist die Rötung weniger deutlich sichtbar. Die Schuppung findet sich ähnlich häufig wie auf heller Haut, kann jedoch gelegentlich statt in der Peripherie auch ungleichmäßig verteilt sein. Die Beurteilung der Gefäße spielt bei der Pityriasis rosea nur eine untergeordnete Rolle [11, 15].

### Dermatitis/Ekzem

Dermatoskopisch findet man eine charakteristische gelbe Schuppung und unregelmäßig verteilte, teils gruppierte Punktgefäße auf gerötetem Hintergrund, wobei die Farbe vom Stadium der Erkrankung abhängig ist. In akut-exsudativen Läsionen sind eher gelbbraune bis dunkelbraune Serokrusten vorherrschend. Im subakuten Stadium zeigt sich eine schwache rote Hintergrundfarbe. Die Schuppung kann weiß oder gelblich-weiß sein, oftmals auch mit einer braunen Färbung und kann vorwiegend peripher gelegen sein. Bei der seborrhoischen Dermatitis zeigt sich ein ähnliches Bild. Beim Handekzem findet man braunorange Schollen, gelbe Schuppen oder Krusten sowie Punktgefäße [3].

### Diskoider Lupus erythematodes

Auch hier sind die Merkmale vom Stadium der Erkrankung abhängig. Im akuten, inflammatorischen Stadium sind follikuläre, keratotische Pfropfe (große, gelbbraune Schollen), Teleangiektasien, perifollikulär angeordnete rote Punkte, ein perifollikulärer weißlicher Hof und weiße Schuppen sichtbar. Die Dermatoskopie kann helfen, die Hautveränderung von einem Lupus pernio oder einem Lupus vulgaris abzugrenzen, da in diesen Fällen die charakteristischen follikulären Veränderungen fehlen. Hingegen sieht man im Spätstadium weiß bis gelbliche strukturlose Areale, Teleangiektasien, Punktgefäße und eine gesprenkelte Pigmentierung. Bei Personen mit dunkler Haut finden sich die gleichen Merkmale und zusätzlich auch häufig braune strukturlose Areale [8]. Des Weiteren gibt es Fallberichte über das Vorkommen eines blauweißen strukturlosen Areals („Schleier“) bei afroamerikanischen Patienten [4].

### Porokeratose

**Figure 3:**
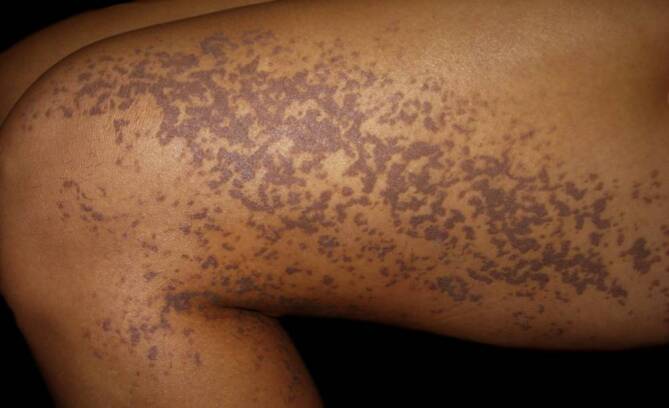

Charakteristisch für die Porokeratose ist die „kornoide Lamelle“ in der Histologie. In der Dermatoskopie zeigt sich diese als ein gut abgrenzbarer, weißgelblicher, ringförmiger hyperkeratotischer Randwall [19]. Dieser kann bei der disseminierten, aktinischen, superfiziellen Porokeratose und auch bei Personen mit dunkler Haut hyperpigmentiert sein. Zusätzlich kann man auch kleine randständige dunkelbraune bis schwarze Schollen finden, die histologisch einer Pigmentinkontinenz und Melanophagen in der papillären Dermis entsprechen [3]. Zusätzlich findet man bei der Porokeratose zentral ein weißes strukturloses Areal und rotbraune Schollen (Abb. 3 und 4; [10]).

**Figure 4:**
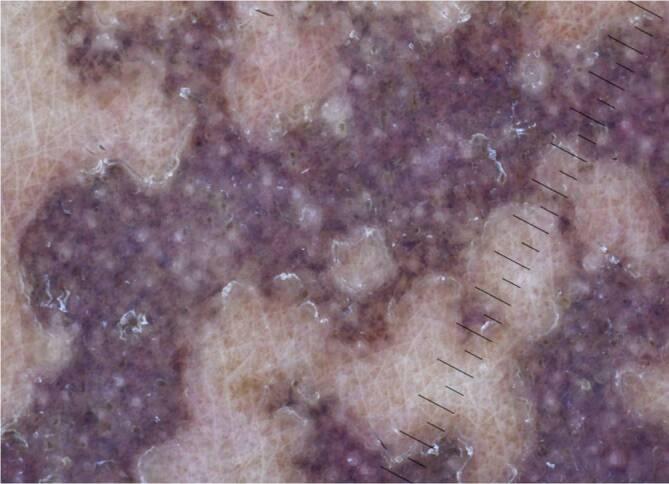

## Pigmentveränderungen

### Melasma

**Figure 5:**
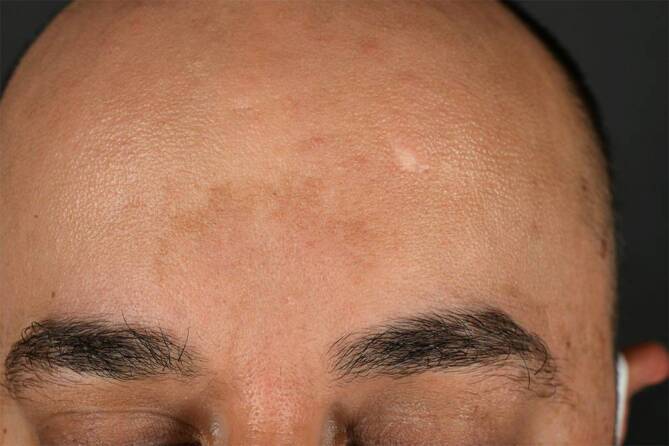

Beim Melasma handelt es sich um eine häufige, erworbene Pigmentstörung, die bevorzugt im Gesicht auftritt und insgesamt häufiger bei Personen mit Hauttyp IV–VI zu finden ist. Da in einigen Fällen klinisch keine eindeutige Diagnose gestellt werden kann, stellt die Dermatoskopie hier ein wichtiges Hilfsmittel dar, um unnötige Biopsien im Gesicht zu reduzieren. Differenzialdiagnostisch kommen hier große solare Lentigines oder ein Lichen planus pigmentosus infrage. Mit dem Dermatoskop zeigt sich interfollikulär ein strukturloses braunes Areal, das histologisch einer Pigmentierung der Basalschicht entspricht (Abb. 5 und 6). In manchen Fällen zeigen sich auch eine graue Pigmentierung mit interostialen Punkten oder aber auch periostale braune Kreise [8].

**Figure 6:**
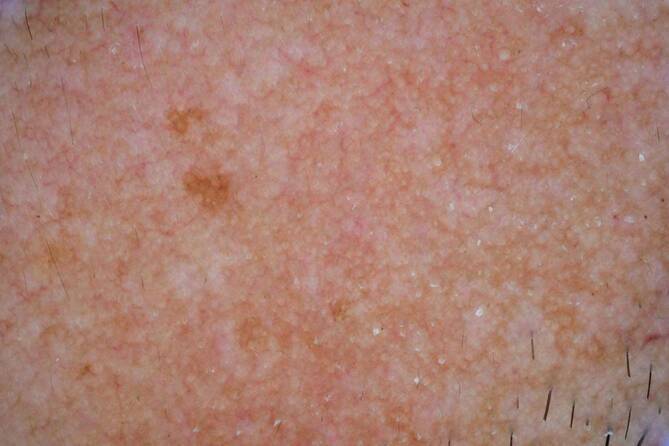

### Lichen planus pigmentosus

Aktuell nimmt man an, dass es sich hierbei um eine seltene Variante des Lichen planus handelt. Im Gegensatz zum Melasma findet man beim Lichen planus pigmentosus kein strukturloses Areal, sondern klassischerweise braune Punkte oder Schollen, die perifollikulär oder linear angeordnet sein können [16].

### Exogene Ochronose

Hierbei handelt es sich um eine iatrogene Dermatose, die vorwiegend bei Personen mit dunkler Haut auftritt. Ursächlich steht die topische Anwendung von Hydrochinon im Vordergrund. Vor allem bei fazialer Hyperpigmentierung ist diese Erkrankung als Differenzialdiagnose zu berücksichtigen. Mit dem Dermatoskop kann man braune bis graue, ring- oder bogenförmig angeordnete Linien und Schollen erkennen. In manchen Fällen zeigen sich auch strukturlos braune Areale, wobei die ausgesparten Follikelöffnungen fehlen [12, 13].

#### Infobox Mehr Informationen zum Thema

- https://dermonaut.meduniwien.ac.at/
- https://dermoscopy-ids.org/

## Fazit für die Praxis

- Die Dermatoskopie hat sich als günstiges und schnell verfügbares diagnostisches Hilfsmittel in der allgemeinen Dermatologie etabliert.
- Bestimmte Dermatosen finden sich fast ausschließlich oder wesentlich häufiger bei Personen mit dunkler Haut.
- Dermatoskopische Basisbegriffe sind bei Personen mit Hauttyp IV–VI ähnlich jenen mit Hauttyp I–III.
- Menschen mit dunkler Haut zeigen ähnliche dermatoskopische Muster wie Menschen mit heller Haut, jedoch können gewisse Strukturen wie etwa die Gefäße schwieriger zu beurteilen sein, während sich follikuläre Veränderungen prominenter darstellen.
- Der Großteil der dermatoskopischen Untersuchungen wurde an Personen mit heller Haut durchgeführt. Weitere Studien mit Personen mit dunkler Haut sind notwendig, um die Dermatoskopie in der allgemeinen Dermatologie in dieser Patientengruppe noch besser nutzen zu können.
